# Mindfulness Reduces Reactivity to Food Cues: Underlying Mechanisms and Applications in Daily Life

**DOI:** 10.1007/s40429-017-0134-2

**Published:** 2017-04-28

**Authors:** Mike Keesman, Henk Aarts, Michael Häfner, Esther K. Papies

**Affiliations:** 1 Department of Psychology, Utrecht University, Heidelberglaan 1, 3854CS Utrecht, The Netherlands; 2 Communication Psychology, Berlin University of the Arts, Berlin, Germany; 3 Institute of Neuroscience and Psychology, University of Glasgow, Glasgow, UK

**Keywords:** Craving, Mindfulness, Decentering, Intervention, Food, Healthy eating

## Abstract

**Purpose of Review:**

Mindfulness-based interventions are becoming increasingly popular as a means to facilitate healthy eating. We suggest that the decentering component of mindfulness, which is the metacognitive insight that all experiences are impermanent, plays an especially important role in such interventions. To facilitate the application of decentering, we address its psychological mechanism to reduce reactivity to food cues, proposing that it makes thoughts and simulations in response to food cues less compelling. We discuss supporting evidence, applications, and challenges for future research.

**Recent Findings:**

Experimental and correlational studies consistently find that the adoption of a decentering perspective reduces subjective cravings, physiological reactivity such as salivation, and unhealthy eating.

**Summary:**

We suggest that the decentering perspective can be adopted in any situation to reduce reactivity to food cues. Considering people’s high exposure to food temptations in daily life, this makes it a powerful tool to empower people to eat healthily.

## Introduction

People are frequently exposed to cues of energy-dense food, which trigger the motivation to eat [1]. As a consequence, even when people have the intention to eat healthily [2], they often fail to do so, as their “obesogenic” environment triggers processes leading to unhealthy eating [3]. Existing psychological interventions show some promise in increasing healthy eating despite these influences [4]. However, they often require changes to be made in the environment, rely on extensive training, work only on specific foods, in specific situations, or are cognitively demanding [4]. Here, we discuss mindfulness as a tool to facilitate healthy eating behavior that may suffer much less from these limitations, along with evidence for its effectiveness and with challenges for further research.

Mindfulness has been suggested to consist of two main components [5]. The first component is well-known in the literature as the regulation of attention to focus and maintain awareness on a stimulus [5]. The second component is the meta-cognitive insight that all experiences are impermanent in nature, rather than them being permanent or reflecting an objective reality [5–7]. This particular quality of attention is often referred to as decentering. Psychological research has frequently focused on decentering as a means to reduce negative affect, such as reducing depression and anxiety, and the construct is also known as “cognitive defusion” or as “teflon mind”, for a review in this domain, see [8]. Adopting this decentering perspective, however, may play a key role in reducing reactivity to both aversive *and* rewarding stimuli alike [9••, 10, 11]. While much recent research shows that multi-component mindfulness-based interventions increase healthy eating [12–14], we suggest that specifically the decentering component of mindfulness plays a key role in contributing to these effects. To make optimal use of a psychological intervention such as mindfulness or decentering more specifically, it is vital to understand how it works [15]. Therefore, the current paper first addresses how food cues lead to unhealthy eating behavior, for example by inducing cravings and other motivational processes, and then discusses the mechanisms through which decentering can reduce such reactivity to food cues. Next, we review empirical evidence of the effects of decentering on, for example, food cravings and food intake. Finally, we discuss how decentering can best be applied to empower people to maintain healthy eating behavior in a world full of temptations and address some challenges for both research and applications.

## The Role of Simulation in Reactivity to Food Cues

To best explain how decentering reduces reactivity to food cues, we need to first outline the mechanism underlying this reactivity. Here, we build on the grounded cognition theory of desire and suggest that perceiving cues of attractive food triggers simulations (“re-experiences”) of consuming the food, which can lead to craving and to motivated behavior, such as choosing and grabbing the food, and physiological responses preparing the body to eat [16–18]. Such simulations can be conceived of as predictions of upcoming situations that are based on earlier experiences and the likelihood of their recurrence [19, 20]. A recent review of fMRI experiments indeed shows that merely seeing a food picture or food word triggers simulations of consumption, such that the brain’s core eating network (involving brain areas that process information such as taste and reward) becomes active [21]. This is especially the case when the food is rewarding, such as when it is energy-dense, or when the participant is hungry. Behavioral studies similarly show that exposure to a rewarding food, in comparison to a neutral food or a non-food object, elicits increased simulations of consumption, such that participants spontaneously think about the taste, hedonic enjoyment, and context of eating the food [22, 23 see also 16]. In addition, fMRI research on humans shows that the extent to which food cues elicit rewarding simulations predicts food consumption and weight-gain over time [24, 25]. Overall, this research provides evidence for the notion that food cues trigger simulations of consumption, which motivate eating behavior.

A process that might further increase the extent to which simulations induce eating behavior is the conscious, vivid elaboration of these simulations [26], which we refer to as immersion [27••]. People can get fully absorbed in a simulation, instead of being aware of the present moment of the actual world. They might for instance think of the hedonic enjoyment of the potential eating experience and how good eating the salty and crunchy chips would make them feel [22]. A recent review of fMRI studies shows that activity in the neural network implicated in creating the experience of immersion is predictive of subjective cravings [28••]. Furthermore, people with lesions in this neural network (e.g., in the posterior cingulate cortex) are better at resisting temptations [28••]. Again, this suggests that rewarding simulations of consumption play a key role in increasing the likelihood of consumption, especially when people get immersed in these simulations.

In addition to inducing cravings, consumption simulations and subsequent immersion in them may also play an important role for inducing bodily preparations to consume, such as salivation [29, 30]. A recent study on the psychological mechanisms underlying salivation to food cues found that when participants were exposed to a food compared to a non-food object, they reported increased consumption simulations, and salivated more, especially when the food was rewarding [23]. Furthermore, participants who were instructed to simulate consumption, compared to participants who were merely exposed to these foods, had increased salivary responses, again especially when the food was rewarding. In related research, cues of rewarding foods, relative to neutral foods, have also been found to induce bodily preparations to eat, such as approach impulses to grab food [31], and the release of the hormone ghrelin, which aids digestion in the stomach and enhances appetite [32, 33]. Overall, these findings suggest that simulation of consumption plays an important role in reactivity to food cues, especially energy-dense food that is typically seen as highly rewarding.

## Decentering Targets the Simulations that Induce Reactivity to Food Cues

As simulations seem to play an important role in inducing reactivity to food cues, it is important to especially target these simulations to decrease the likelihood of unhealthy eating. We suggest that decentering can be a useful tool to do so. When adopting the perspective of decentering, all experiences such as thoughts and simulations are conceived of as impermanent states of one’s own mind, rather than of the world, making them less compelling. Furthermore, by observing these experiences as events that arise and dissipate again, elaboration and immersion in these experiences are reduced [34]. As an example, consider having just come home from a long day of work, sitting on the couch, and watching TV. If you often eat chips in such a situation, this may trigger simulations of consumption, for instance consisting of the taste of chips, grabbing them, and the hedonic enjoyment of eating. This process may then induce all sorts of reactivity, such as thoughts of consumption and cravings to eat chips. Being immersed in these experiences and continuing to elaborate on them, such as on the potentially good feeling of eating the chips, further increases this reactivity [26, 28••], such that you may find yourself wandering to the kitchen in search of a bag of chips. Conversely, when adopting the perspective of decentering towards these experiences, you observe them as no more than impermanent mental events, making them less vivid and compelling. Thus, any thoughts such as about the taste and texture of the food, the pleasure of eating, and the rewarding feeling of satiation are observed as mere passing mental events. As a result, elaboration and immersion are not precipitated, and consumption and reward simulations become less compelling to act on. In this way, decentering can work to prevent cravings, bodily preparations to eat, and actual consumption.

Recent research provides important evidence for the notion that decentering reduces reactivity through decreased immersion and elaboration, albeit in domains other than food and eating behavior. In an fMRI experiment on craving for cigarettes, for instance, participants who adopted a decentering perspective had reduced activity in the neural network corresponding to craving, compared to control participants [35]. Importantly, functional connectivity between prefrontal and craving-related areas was not increased, suggesting the absence of top-down regulation. Instead, several fMRI experiments show that the adoption of a decentering perspective leads to reduced activity in the neural network corresponding to immersion [34, 36–38]. In addition, an fMRI experiment on pain processing provides an excellent example of how reduced reactivity to pain is achieved through decentering [39]. Zen meditators, whose mindset is heavily focused on adopting a decentering perspective [40], had reduced reactivity to painful heat stimulations compared to non-mediators, even though they showed increased activity in the neural network corresponding to sensory and pain processing. A decoupling of this network from prefrontal areas corresponding to cognitive elaboration and engagement, however, was associated with the reduced reactivity to the painful stimulation. Again, this suggests that decentering does not lead to extensive top-down downregulation of experiences, but rather works to reduce reactivity in the first place by decreasing cognitive elaboration on and immersion in experiences.

## Decentering Reduces Reactivity to Food Cues

Decentering may be an especially effective tool for reducing reactivity to food cues because it targets its underlying mechanism, namely vivid and compelling consumption and reward simulations. Indeed, several lines of evidence have recently shown that decentering inductions among non-meditators can affect responses to food cues [31, 41••, 42]. Such decentering inductions for non-meditators typically take anywhere from 3 to 15 min and focus on the insight that experiences are merely impermanent events that arise and dissipate again. They also often include a metaphor to portray how this might work, such as looking at a flowing river rather than being carried away by it, or being the driver of a “mindbus” with thoughts as the passengers that hop on and after some time, hop off again. In carefully designed experiments, participants are then instructed to adopt this decentering perspective or a control perspective towards any experience that a food cue might elicit.

In one line of studies [27••, 31], participants were instructed to adopt a decentering perspective, or a control perspective, to any thoughts and experiences elicited by images of energy-dense snacks. The control perspective consisted of instructions to immerse oneself fully in the presented images, of instructions to view the images in a very relaxed manner, or control participants received no intervention at all. Adopting a decentering perspective to the experiences elicited by the images was expected to make them less compelling and thus decrease the reward expected from them. Future encounters with these snacks should then elicit less reactivity as a consequence. Indeed, in three experiments, subsequent exposure to images of energy-dense snacks no longer elicited approach impulses in participants in the decentering condition, whereas participants in the control condition still exhibited them [31]. In addition, decentering also reduced hypothetical unhealthy food choices in a laboratory task, experienced cravings, and actual unhealthy food choices in a buffet setting [27••]. Here, decentering participants chose to eat fewer unhealthy snacks and more salads than control participants, and their entire lunch consisted of fewer calories [27••]. These experiments thus show that decentering can effectively reduce reactivity to food cues, such that approach impulses, cravings, and unhealthy food choices are less likely to ensue.

Decentering can also be adopted in the heat of the moment, for example in a situation where one is tempted to eat. For instance, in several recent experiments, participants were instructed to adopt a decentering perspective towards any of their food-related thoughts or to adopt a control perspective. When decentering participants were then reminded of an energy-dense snack, or when an attractive snack was put in front of them, they reported reduced cravings. Furthermore, they also displayed a reduced salivary response, suggesting reduced bodily preparations to eat [43, see also 42]. Another set of studies has demonstrated further effects of decentering on actual eating behavior outside the laboratory, such as reducing chocolate consumption in participants’ natural environment, over a period of 5 and 7 days, respectively [41••, 44]. In sum, these experiments show that a decentering perspective can successfully be adopted towards food temptations, which then reduces reactivity and actual consumption.

As most skills improve with extended practice [45, 46], the skill of adopting a decentering perspective should also be enhanced by adopting it repeatedly. A recent experience sampling study indeed found that repeated practice of mindfulness meditation by a meditation naïve sample led to increased adoption of decentering in daily life [47]. Furthermore, a recent correlational study on decentering and food cravings found that among active meditators, the amount of meditation practice—which typically includes elements of decentering—predicted of the adoption of a decentering perspective in daily life [48]. Importantly, the extent to which these meditators adopted a decentering perspective towards their thoughts in turn predicted decreased food cravings in daily life. Mere awareness of these thoughts, however, did not predict reduced cravings [48]; for similar findings, see [49]. Overall, extended practice of adopting a decentering perspective may facilitate its adoption in daily life, which may then reduce the reactivity to food cues that one encounters throughout the day.

## Benefits of Using Decentering as a Tool to Reduce Reactivity to Food Cues

Decentering targets the psychological process of simulation that underlies reactivity to food cues. We suggest that this approach to preventing food cue reactivity has many benefits over perhaps more traditional approaches to facilitating healthy eating. One important benefit is that by targeting consumption simulations, decentering may prevent potent cravings from arising in the first place. In addition, adopting a decentering perspective before a craving has fully developed may be less cognitively demanding than coping with a full-blown craving, especially when in a situation where tempting foods are highly accessible and eating and reward simulations can easily be elaborated on. Furthermore, targeting consumption simulations diminishes not only cravings but also other reactivity that may facilitate consumption, such as approach impulses. A further benefit is that in situations in which a health goal may not be at the forefront of one’s mind, a decentering perspective would still help to reduce the risk of unhealthy intake. This is important as in many such daily situations, food temptations are present, for example when going out with others, when passing the candy bowl in the office, or when returning home after a long day at work. Previous interventions have been shown to effectively activate health goals in situations where eating decision are made, such as in a supermarket [50, 51]. As food reactivity may work to subsequently overpower and inhibit these health goals [52], decentering may work well to complement such interventions. In sum, we suggest that decentering can be a powerful tool to facilitate a lifestyle of healthy eating precisely because it targets the simulations and thoughts that underlie reactivity to the obesogenic environment.

Another benefit of decentering is its broad applicability. As all experiences are impermanent in nature, decentering can be applied to any experience, and in any situation—as long as one remembers to do so [53••]. Moreover, the benefits of decentering in daily life have been found to materialize in multiple domains. Among meditators, for instance, adopting a decentering perspective was associated with both reduced food cravings and increased resilience to stressful events [48]. Decentering has also been found to underlie the effects of mindfulness trainings to reduce depression and anxiety [54, 55]. As a result, decentering could potentially not only counter cravings from food cues, such as in the supermarket or from food ads, but may also counter cravings stemming from stressors, such as a heavy workload. Decentering has also been shown to reduce cravings for other substances, such as for alcohol and cigarettes, helping to maintain an overall healthy lifestyle [35, 53••, 56, 57]. Thus, decentering can be broadly applied to reduce reactivity in various circumstances, towards various rewarding stimuli, and may thereby help people to attain better health outcomes.

Decentering can also be applied in different forms. For instance, during counseling sessions, decentering might be applied towards energy-dense foods, or their images [58]. As a result of this approach, future encounters with this food may lead to reduced reactivity, making it easier not to eat it. People with strong habits of unhealthy eating and people who are obese might especially benefit from applying decentering in such controlled settings, as they often exhibit particularly strong reactivity to food cues that make it difficult for them to reduce reactivity in the heat of temptation [59]. For others, however, it might be especially useful to adopt a decentering perspective towards the experiences that are elicited by food cues in the heat of the moment, in daily life, e.g., [41••]. Possibly, being taught the perspective of decentering only once and realizing the impermanent nature of one’s food-related thoughts may allow one to adopt this stance independently and without further instruction in any tempting situation. This could empower people to eat healthily despite food temptations in the environment. This option may require significant cognitive capacity at first, because it may be effortful or difficult to apply and maintain this perspective. Repeated practice, however, would make it less effortful on future occasions as extended practice leads to increasingly automated execution [60]. Indeed, evidence so far suggests that repeated meditative practice facilitates the adoption of a decentering perspective in daily life, with beneficial consequences for health behavior [47, 48].

## Applications and Challenges for Future Research

Although decentering seems to have great potential for reducing reactivity to food cues, research in this area is still relatively limited, especially considering the vast literature on mindfulness more generally, for reviews, see [58, 61, 62]. For decentering to reach its maximum potential as a tool to help people eat healthily, it is important to focus on how it can best be taught and applied towards food cues. In many Buddhist contemplative traditions, people both meditate and receive instruction from highly experienced teachers, and many Western mindfulness-based interventions derived from them take a similar approach, such as the standardized 8-week course of Mindfulness-Based Stress Reduction MBSR [63]. Developing decentering through meditation has the benefit that the first component of mindfulness, attention regulation, is typically also developed. In meditation, for instance, one may have the repeated experience of becoming aware of distractions and bringing attention back to a focal object [64]. Training this component of mindfulness increases the likelihood of experiences reaching awareness, which may facilitate the effective application of a decentering perspective, because one may be more likely to notice food-related thoughts and cravings arising at a very early stage [6, 53••]. The ability to regulate attention may furthermore facilitate body awareness, such as of satiety, or increase awareness of one’s behavior being instigated by habit rather than reflective choice, and thereby assist people to make fewer impulsive eating choices [12, 14]. The traditional path of developing decentering through meditation may thus offer additional benefits compared to learning decentering in a “stand-alone manner” for enhancing healthy eating.

For some people, it may be more attractive to develop decentering without meditation, such as by direct instruction, as was done in most of the experiments among non-meditators addressed in the current review. Such an instruction is brief, yet still effective, whereas standardized mindfulness-based interventions such as MBSR typically assign participants to 45 min of meditation per day for a period of 8 weeks [63]. Moving from no meditation experience to 45 min of meditation per day may be cognitively demanding and may also be difficult to implement in daily life. Furthermore, some people may be unwilling to meditate, as they hold preconceived notions of meditation being “airy-fairy” [65], rather than being evidence-based. We suggest that the potential drawback of not developing the first component of mindfulness may be offset by combining an intervention using decentering instructions with the monitoring of eating behavior, such as by keeping a food diary. Future longitudinal research on the brief decentering instruction and eating behavior, especially in combination with interventions that enhance awareness of eating behavior [66], would increase our understanding of the process of successfully adopting a decentering perspective. For now, it seems promising that decentering can be developed both through meditation and by using instructions that do not require meditation.

A potential concern regarding decentering as an intervention is its decontextualized use in the West as compared to within a Buddhist ethical framework [67, 68]. From a practical perspective, this may lead to the suboptimal application of decentering and to undesirable outcomes. It is important, for instance, to not conflate decentering with a license for apathy merely because it reduces immediate reactivity to food cues. Becoming less influenced by environmental food cues through decentering should rather be used as an opportunity to act in line with one’s goals, such as eating healthily. Furthermore, while decentering may help to deal with set-backs, such as an occasional violation of one’s health goals, it should not be conflated with non-judgment [6] or be used as a way to not discriminate between healthy and unhealthy eating. Indeed, the mere acceptance of food-related thoughts without deconstructing them through decentering has been shown to actually increase, rather than decrease, food cravings [69]. Decentering is also distinct from taking distance from one’s thoughts. It is the insight that one’s thoughts, whatever they are, are impermanent in nature, rather than being permanent or reflecting an objective reality. Distancing, however, does not reduce reactivity to concrete situations but reduces reactivity to experiences that are deliberately analyzed from a broader “why” perspective [70, 71]. For people to optimally benefit from adopting a decentering perspective, then, it is important that they are provided with a clear instruction of what it is, and how to best apply it to reduce reactivity to food cues, such as when decentering is developed within a Buddhist ethical framework.

## Conclusion

Decentering is the metacognitive insight that all experiences are impermanent. This perspective can be learned through meditation, but non-meditators can also adopt it through brief non-meditative decentering instructions. In various well-controlled experiments, decentering has been found to consistently and effectively reduce reactivity in the forms of approach impulses, subjective cravings, and actual consumption of energy-dense foods. We suggest that decentering is a powerful tool to reduce reactivity because it directly targets the simulations and immersion underlying this reactivity. One added benefit of decentering as a tool to reduce unhealthy eating is that it is broadly applicable, towards any stimulus, and in any situation. Furthermore, as the adoption of decentering can be considered a skill, repeated practice further facilitates its application in daily life, which may underlie the effectiveness of more comprehensive mindfulness interventions that typically include a decentering element. In this world full of tempting yet unhealthy foods, we suggest that decentering can play an important role in empowering people to eat healthily.
